# The Mediating Role of Engagement on the Achievement and Quality of Life of University Students

**DOI:** 10.3390/ijerph18126586

**Published:** 2021-06-18

**Authors:** Inmaculada García-Martínez, José María Augusto Landa, Samuel P. León

**Affiliations:** 1 Department of Didactics and School Organization, University of Granada, 18071 Granada, Spain; igmartinez@ugr.es; 2 Department of Psychology, University of Jaén, 23071 Jaén, Spain; 3 Department of Pedagogy, University of Jaén, 23071 Jaén, Spain

**Keywords:** academic engagement, emotional intelligence, resilience, quality of life, university students

## Abstract

(1) Background: Academic engagement has been reported in the literature as an important factor in the academic achievement of university students. Other factors such as emotional intelligence (EI) and resilience have also been related to students’ performance and quality of life. The present study has two clearly delimited and interrelated objectives. First, to study the mediational role that engagement plays in the relationship between EI and resilience on quality of life. Secondly, and similarly, to study the mediational role of engagement in the relationship between EI and resilience, but in this case on academic achievement. (2) Methods: For this purpose, four scales frequently used in the literature to measure emotional intelligence, resilience, academic engagement and quality of life were administered to 427 students of the University of Jaén undertaking education degrees. In addition, students were asked to indicate their current average mark as a measure of academic performance. Two mediational models based on structural equations were proposed to analyse the relationships between the proposed variables. (3) Results: The results obtained showed that emotional intelligence and resilience directly predicted students’ life satisfaction, but this direct relationship did not result in academic performance. In addition, and assuming a finding not found so far, engagement was shown to exert an indirect mediational role for both life satisfaction and academic performance of students. (4) Conclusions: The findings of the study support the importance of engagement in the design and development of instructional processes, as well as in the implementation of any initiative.

## 1. Introduction

The academic achievement of students has been a constant concern for educational research. Studies on effectiveness in educational contexts have focused on identifying the factors that can best predict achievement in order to provide early intervention in the education process and reduce academic failure. International organisations such as the OECD [1] and international research have identified teacher performance as the primary external influencing factor in the analysis of student achievement [2,3]. Other studies have examined some internal factors that may affect the study of academic performance, such as intelligence and personality traits [4,5]. Some point to a relationship between life satisfaction and academic achievement [6], while others do not support this correlation [7]. However, life satisfaction is related to other variables, such as perceived academic ability, positive attitudes towards school, and school appreciation [8].

Studies on life satisfaction have mostly been situated in the general context of subjective well-being research [9,10]. This concept of subjective well-being includes two clearly differentiated components that have followed parallel lines of research: firstly, cognitive judgments (judgments or evaluations that people make about their lives) about life satisfaction [11]; and, secondly, affective evaluations (the pleasure experienced by people with their most frequent feelings, emotions and states of mind) about mood and emotions [12,13]. The present study addresses the analysis of the cognitive component of subjective well-being. More specifically, it will examine one of the components of psychological well-being, life satisfaction, which refers to the cognitive evaluation of a person’s quality of life according to his or her own standards [14]. Thus, life satisfaction is defined as a person’s overall assessment of his/her life, comparing what he/she has achieved, and his/her expectations [15,16]. Feeling good about one’s life is a primary concern for most people [17]. Generally speaking, life satisfaction is associated with people who are able to stay motivated, regardless of the challenges or adverse situations they face in their daily lives. This ability regulates their negative thoughts, enables them to empathise with others and it keeps them optimistic [18]. Studies have shown the importance of life satisfaction among university students as a strength that facilitates their adaptive development [19].

Several studies have found that life satisfaction is closely related to job occupation, education, financial resources, leisure and family [20,21,22]. It can be concluded that life satisfaction impacts on an individual’s social life and psychological behaviour [23]. Some studies have found that life satisfaction is an important predictor of university students’ engagement in academic performance [24]. Thus, university students with high life satisfaction tend to report greater satisfaction with their academic experiences. Associations have been found between life satisfaction and positive academic expectations, academic self-efficacy, greater perceived progress towards goals, and lower levels of academic stress [25,26].

Below, it reviews a set of personal resources and/or strengths of university students that play an important role in explaining academic achievement and quality of life within this group. Among these resources, the focus will be on emotional intelligence (EI), resilience, and academic engagement. Taking into account that achievement, as explained above, has been predicted by multiple factors, an attempt will be made to explain the achievement variable as well as life satisfaction in university students through these strengths to which it has been mentioned.

### 1.1. Emotional Intelligence

The most widely accepted conceptualisation of emotional intelligence defines it as an ability to perceive, assimilate, understand and regulate its personal and other people’s emotions [27]. According to this model, it emphasises the importance that knowledge and regulation of emotions may have on the personal, occupational and social well-being of individuals. The EI is understood as the ability to process the information provided by emotions [28]. Emotionally intelligent people are those who know how to attend to the emotions that arise in their environment, they are capable of understand the possible causes and consequences of these emotions and, consequently, they have the ability to develop the strategies needed to regulate or manage their emotions [27,28]. Extensive research has shown the relationships between EI and subjective well-being in different groups [29,30,31,32,33]. According to these studies, the emotional skills that include the EI construct help to make better use of emotional information and improve life satisfaction of people [27].

Several studies have found a positive relationship between EI and academic achievement [34,35,36]. A recent meta-analysis conducted by MacCann [4] has shown that EI is the third most important predictor after intelligence and conscientiousness for academic achievement. The authors also propose three mechanisms that underlie the EI and academic achievement link: (a) regulating academic emotions, (b) building social relationships in the school/university context, and (c) academic content overlaps with EI.

### 1.2. Resilience

Psychological resilience is generally defined as the ability of individuals to adapt to stressful and challenging situations. It is also conceptualised as a stress-resistant attitude and it represents the ability to cope with adverse situations [37]. Resilience is used to label people who are able to easily and quickly overcome their life-related mishaps and career aspirations [38]. Resilience has been negatively associated with psychological distress in university students [39], suggesting that it may help them adapt to the new context’s performance. Therefore, it is very important to promote resilience among university students in order to cope with their difficulties and to understand the mechanisms where resilience affects functioning and positive change. Resilience is considered an important construct in positive psychology, and it plays an important role in one’s life satisfaction [40]. Thus, several studies have shown that resilience is related to high levels of life satisfaction [41,42,43]. There is evidence that resilient students have skills that allow them to feel that they are capable of achieving their goals, that they have control over their lives, and they are able to see themselves as responsible for their own outcomes [44]. Resilient learners also show higher levels of resourcefulness and they tend to be optimistic about achieving their predetermined goals, as optimism is associated with expected outcomes [45]. There is existing literature which shows the relationship between high resilience and good academic performance [46,47,48].

### 1.3. Academic Engagement

Engagement in the field of education corresponds to academic engagement, which refers to a state of psychological well-being and commitment to studies [49]. Academic engagement is defined as a state of psychological well-being composed of three dimensions (vigour, dedication, and absorption) of intrinsic commitment to studies [50]. Thus, the vigour dimension is understood as the willingness to devote effort to a specific action and to persist despite difficulties. Secondly, the dedication dimension is characterised by involvement in the task and the experience of pride and enthusiasm for it. Finally, the absorption dimension refers to concentration and immersion in the action itself. Studies in educational contexts have shown that academic engagement is related to self-efficacy and self-esteem, foster academic engagement and positively impact on students, such as high achievement and satisfaction [51]. Academic engagement is also negatively associated with school burnout, study demands, and depressive symptoms [52]. Studies have found that academic engagement produces positive effects on academic factors such as improved academic performance [53,54]. There are many studies linking positive engagement outcomes with life satisfaction [55,56]. Moreover, longitudinal studies with adolescents have shown that increased life satisfaction has been associated with academic engagement [57,58].

### 1.4. Objectives of the Study

The interest in positive psychology and its applications in educational settings has grown exponentially in recent years [59]. In relation to the previous results presented in the theoretical sections, our study is focusing on investigating the role of “strengths” on quality of life and academic achievement in university students. Engagement is one of the mechanisms that plays a mediating role in the relationship established between different variables [60], including professional adaptability and self-regulation [61], self-efficacy [62], and emotional intelligence [63], among others.

**Figure 1:**
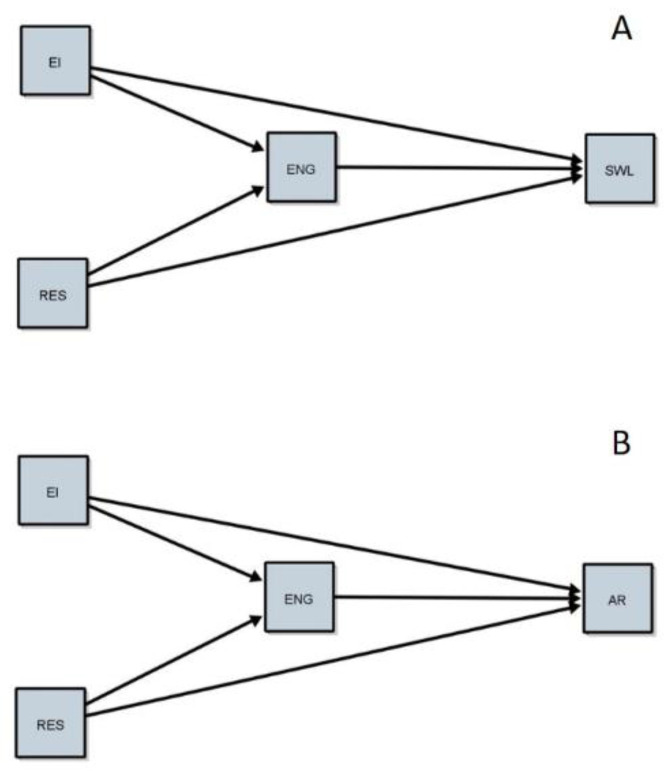
Mediational SEM models. Panel (**A**) for Satisfaction with Live as VD and Academic Record for Panel (**B**). EI = Emotional intelligent; RES = Resilience; ENG = Engagement; SWL = Satisfaction with Life; AR = Academic Record.

Specifically, it sets out two main objectives in this research:

1. To study the mediational role of engagement in the relationship between EI and resilience on quality of life. Specifically, we hypothesize that EI and resilience will explain part of the quality-of-life relationships directly and indirectly through their relationship with engagement (see Figure 1A).
2. To study the mediational role of engagement in the relationship between EI and resilience on academic achievement. Specifically, we hypothesize that EI and resilience will explain part of the academic achievement relationships directly and indirectly through their relationship with engagement (see Figure 1B).

## 2. Materials and Methods

### 2.1. Participants

The study was carried out with 427 students from the University of Jaén (Spain), with an average age of 21.50 years (SD 3.61). Of the total, 88.3% were female and 11.7% were male. These percentages are proportional to the distribution of males and females in the total population of students undertaking ed in Spain [64]. Regarding the distribution of students by academic degree, we found that 74.5% belonged to the Degree in Early Childhood Education, 16.6% to the Degree in Social Education, and 8.9% to the Degree in Primary Education. All students participated on a voluntary basis.

Previously, the minimal sample size was calculated at 95% confidence level, with a 5% confidence interval at 80% of statistical power. The estimated minimum sample size was 385. According to Hair et al. [65], the general rule to calculate the minimum sample size for factor treatment in a survey is to have a minimum of 5 observations per variable (5:1). In the current study, the scales consisted of 43 items, so the minimum for the factorial treatment would be 215.

### 2.2. Instruments

#### 2.2.1. Emotional Intelligence

Wong and Law Emotional Intelligence Scale [66]:

This scale is composed of 16 short sentences used to evaluate four dimensions: self-emotion appraisal (SEA), other’s emotion appraisal (OEA), use of emotion (UOE) and regulation of emotion (ROE). Respondents are asked to rate their agreement with the sentences on a five-point Likert scale ranging from 1 (strongly disagree) to 5 (strongly agree). We used the Spanish version [67] which has shown adequate validity and reliability in Spanish contexts (α = 0.91).

#### 2.2.2. Resilience

Resilience Scale (RS-14) [68,69,70]:

This instrument is designed to assess the level of individual resilience through Equanimity, which refers to a balanced perspective on life and experiences, and could be seen as the ability to accept and adjust to circumstances as they arise, thus moderating extreme responses to adversity, a construct often related to mood. RS-14 scale validated by Sánchez-Teruel and Robles-Bello [71] was used to determine resilience, which consists of 14 items, distributed in two dimensions: (a) personal competence and (b) self-acceptance and life acceptance. The reliability analysis of the scale was α = 0.93.

#### 2.2.3. Student Engagement

Utrech Work Engagement Scale for Students (UWESS-9) [72]:

The instrument has 9 items measuring the dimensions of vigour (i.e., My tasks as a student make me feel energised), dedication (i.e., I am excited about my studies) and absorption (i.e., I am immersed in my studies) to studies. The response alternatives are presented in a 6-point Likert format (0 = not at all, 6 = every day). The reliability of the instrument is good in its adaptation to the Spanish context. In fact, the Cronbach alpha was 0.84 for vigour, 0.89 for decision making and 0.79 for absorption.

#### 2.2.4. Quality of Life

Quality of Life Scale [15]:

This is a five-item inventory that measures the satisfaction with life, with questions such as “In most cases my life is close to my ideal” and “I am satisfied with my life”. The SWLS was designed as a 7-point Likert-type scale, whose response range is from 1 (strongly disagree) to 7 (strongly agree). The Spanish version was adapted by Díaz Morales [73], with a Cronbach alpha of 0.87.

#### 2.2.5. Academic Achievement

In order to measure students’ academic achievement, we asked for their average mark to date of their degree (overall average mark obtained in the course by the student when asked).

### 2.3. Procedure

The instruments were administered using Google Form tool (Google inc. LLC., Menlo Park, CA, USA). The researchers attended the classes to find potential participants and to ask permission for participation and then, researchers explained the purpose of the research. The researchers were present while the participants completed the questionnaire and answered any potential questions they had. They were also provided with the researchers’ emails in case they had any further questions. Participation in the study was completely voluntary, according tothe Declaration of Helsinki in 1975 and its next adjustment from Brazil in 2013 [74], respecting the national legislation for clinical trials (223/2004 Law from February 6th), biomedical research (14/2007 Law from July 3rd), and participant’s confidentiality (15/1999 from December 13th) and the Human Research Ethics Committee of the University of Jaén, regulated by Andalusian Decree 439/2010 of December 14th.

### 2.4. Data Analysis

All analysis in this study was conducted with the R software (R Core Team, Vienna, Austria). The α value for all statistical tests was set to 0.05. Data screening was performed before the factorial analysis to evaluate the distribution of data and assumptions. Before the treatment of the data obtained with the scales, the validity and internal consistency of the scales was verified by confirmatory factor analysis. Confirmatory factorial analysis (CFA) and SEM model analysis were conducted with lavaan R package [75]. Diagonally weighted least squares (DWLS) was used as estimation method for CFA [76] to account multivariate non-normality. Cronbach’s alpha and McDonald ω were used to assess reliability [77]. Once the factorial treatment had been performed with the results of the scales, the scores given by the participants were measured by the factor load resulting from the CFA, that is, the raw scores given by the participants to each item were multiplied by the standardized factor loads of each item [78]. Two theoretical mediational models based on structural equations (SEM) are proposed: one where the dependent variable was life satisfaction (SWL) and the other where the dependent variable was academic performance (AR).

## 3. Results

Prior to the factorial treatment, the distribution of the data and the assumptions for the factorial treatment were analysed. The results of Mardia’s Multivariate Normality Test showed that the data did not maintain a multivariate normal distribution (ZKurtosis 51.229, *p* < 0.01). Data screening showed that the linearity assumption was not fulfilled as no item showed multicollinearity (r > 0.90), nor singularity (r > 0.95). To analyse the homogeneity and homoscedasticity assumptions, it was analysed the residuals resulting from the regression between our data and randomly generated data. Any anomalies in the distribution of the residuals were due to the behaviour of our data [79]. The resulting distribution was not violating any assumptions, showing a distribution of standardized regression residuals mostly between −2 and +2.

### 3.1. Analysis of the Subscales

Before using the scores obtained by the different scales, it was examined the psychometric properties of the data obtained with the different scales. Table 1 indicates the results of the confirmatory factorial analysis (CFA) and the reliability study for each of the scales used in this paper. As can be seen, all scales showed excellent levels of validity [65] and good levels of reliability.

### 3.2. Mediational Models

After analysing the data obtained by our scales and verifying that they offer acceptable levels of validity and reliability, the direct and indirect relationships of the proposed mediational models were analysed. Figure 1 shows the two proposed mediational models in panels A and B. The rectangular figures represent the scores for each latent variable obtained through the scales, and the one-way arrows show regression relationships between the factors.

Panel A (Top) shows the relationship of the engagement measure (ENG) between emotional intelligence (EI) and resilience (RES) with life satisfaction (SWL). Panel B (Lower) shows a similar mediation model to the previous one, except that the dependent variable in this case is the students’ academic performance measured by the academic record (AR). Results of the relationships of the different relationships proposed in the mediational models are presented in Table 2. As can be seen, in the model for SWL (Panel A), all the relationships analysed were found to be significant. This suggests that the predictor variables EI and RES in the model are directly related to SWL, and also indirectly through the mediation of ENG. In the case of academic performance (panel B), except for the direct relationship between EI and RES with AR (β = 0.13, and β = −0.15 respectively), and the total relationship of RES and AR (β = −0.10), all relationships were found to be significant. These results indicate that engagement plays a mediating role between EI and RES and academic performance, with no clear direct relationship between these variables.

## 4. Discussion

Results of the present research have shown significant relationships between personal strengths (EI, resilience, and engagement) and the criterion variables (quality of life and academic achievement). More specifically, model A (Figure 1A) showed a direct relationship between EI and life satisfaction, and previous studies have shown similar results with university students [30,42,80]. Resilience has shown a direct relationship with quality of life, explaining part of the variance of the latter. These data are also in line with previous studies [81,82], which, like our results, highlight the importance of resilience in promoting quality of life. As for the measurement of academic engagement in the relationship between EI and resilience with quality of life, a partial indirect mediation was found. That is, EI and resilience explained part of the variance in quality of life through academic engagement. These findings suggest that the model shows partial mediation. Moreover, it is confirmed that the quality of life depends on the ability of university students to perceive, facilitate, understand, regulate their own and other people’s emotions and that they must also be able to overcome problems related to their lives.

Regarding model B (Figure 1), no direct relationships were found between EI and resilience with academic achievement (although in the case of resilience it is very close to significance). However, indirect relationships were found. It means that EI and resilience predict academic achievement indirectly through academic engagement. Moreover, it indicates that the model shows a fully mediated relationship. This confirmed that academic achievement depends on the degree of academic engagement, although academic engagement is related to the subject’s ability to perceive, facilitate, understand, and regulate their own and others’ emotions, as well as being able to easily and quickly overcome setbacks related to their life and career aspirations.

Our results are in accordance with those of Urquijo and Extremera [83], who analysed the mediation of academic engagement in the relationship between EI and academic satisfaction. These authors, as we have, found no direct relationship between EI and academic satisfaction but an indirect relationship between EI and academic satisfaction through the mediation of academic engagement.

### 4.1. Limitations of the Study

In terms of limitations, one of them it refers to our design. It is a cross-sectional design which does not allow to establish a causal relationship between the variables under consideration. Further research on this topic should consider and evaluate mediational models through a longitudinal design, in order to gain a better understanding of the associations between these variables. Another limitation refers to the use of self-reports to assess variables leading to a multicollinear effect. In this study, SEM was used to reduce such effects, but it would still be advisable for future studies to use performance or execution means to obtain more information about possible differences in the relationships between variables and reduce subjectivity at the same time. Another limitation is the composition of the sample. Further studies should consider the use of samples from different universities and degrees in order to be able to generalise the results. Finally, the scarcity of studies carried out with university and higher education students that analyse and contrast the mediational effect of academic engagement in the relationship between EI and resilience on quality of life and academic achievement (in fact, to our knowledge, this is the only study to date that has taken these variables together into account) is another limitation when it comes to supporting and contrasting the results obtained.

### 4.2. Practical Implications

The European Higher Education Area (EHEA) is based on a reorganisation of knowledge, an emergence of new expectations, an internationalisation of education, and real links between business and universities. It is in this construction of a new vision where special importance is given to not limiting oneself to purely academic and cognitive knowledge, but rather to develop other skills such as emotional intelligence and resilience in order to be able to face new challenges of adaptation. Given the overall observed benefits of the connection between EI, resilience, and academic engagement for both life satisfaction and school achievement, the implementation of EI and resilience interventions should be considered a priority for university students.

Several studies argue that emotional intelligence may be modified through programmes and experiences [84,85,86]. The results found in this study support the idea of considering interventions in emotional intelligence with a view to improving engagement through programmes that help to enhance academic achievement and quality of life. In addition, workshops or training programmes in resilience skills would make students better able to overcome adversity and experience better adjustment and well-being when they face challenges or difficulties. Roberson et al. [87] argue that resilience intervention protocols have produced adaptive changes in several outcome variables (e.g., well-being, performance).

The combination of both types of training would result in an improvement in academic engagement and thus in the academic achievement and quality of life of university students.

## 5. Conclusions

This study provides empirical evidence of the importance of EI, resilience and engagement in predicting life satisfaction and academic achievement.

This study argues for the importance of providing students with essential tools in order to facilitate their adaptation to the university environment. In this manner, the importance of EI, resilience, and positive psychology elements such as academic engagement in higher education is highlighted. Furthermore, this study is truly innovative in that it also analyses the importance of EI and resilience on academic engagement, which influences the quality of life of university students.

## Figures and Tables

**Table 1:** CFA Results and Reliability for the Different Scales.

								90% CI			
Scale	*χ* ^2^	df	*p*	CFI	TLI	SRMR	RMSEA	Lower	Upper	α	ω
WLEIS-S	79.623	84	0.615	0.999	0.999	0.046	0.000	0.000	0.024	0.851	0.857
RS-14	144.873	77	<0.001	0.982	0.978	0.068	0.045	0.034	0.057	0.882	0.887
SWLS	2.004	5	0.849	0.999	0.999	0.021	0.000	0.000	0.037	0.833	0.835
UWES-9	18.089	24	0.799	0.999	0.999	0.044	0.000	0.000	0.026	0.832	0.831

Note. WLEI-S = Wong and Law Emotional Intelligence Scale; RS-14 = Resilience Scale; SWLS = Satisfaction with Life Scale; UWES-9 = Utrech Work Engagement Scale for Students.

**Table 2:** Indirect and Total Effects in Mediational Model.

					95% C.I.				
Model	Type	Effect	Estimate	SE	Lower	Upper	*β*	*z*	*p*
A	Indirect	EI ⇒ ENG ⇒ SWL	0.06	0.02	0.01	0.10	0.04	2.58	0.010
		RES ⇒ ENG ⇒ SWL	0.08	0.03	0.02	0.13	0.05	2.72	0.007
	Component	EI ⇒ ENG	0.27	0.09	0.10	0.45	0.22	3.11	0.002
		ENG ⇒ SWL	0.22	0.05	0.13	0.31	0.20	4.62	<0.001
		RES ⇒ ENG	0.36	0.11	0.15	0.56	0.24	3.36	<0.001
	Direct	EI ⇒ SWL	0.25	0.09	0.08	0.42	0.18	2.84	0.004
		RES ⇒ SWL	0.46	0.10	0.25	0.66	0.28	4.38	<0.001
	Total	EI ⇒ SWL	0.31	0.09	0.13	0.48	0.23	3.48	<0.001
		RES ⇒ SWL	0.53	0.11	0.33	0.74	0.33	5.06	<0.001
B	Indirect	EI ⇒ ENG ⇒ AR	0.06	0.03	0.01	0.12	0.04	2.42	0.015
		RES ⇒ ENG ⇒ AR	0.08	0.03	0.02	0.15	0.05	2.54	0.011
	Component	EI ⇒ ENG	0.27	0.09	0.10	0.45	0.22	3.11	0.002
		ENG ⇒ AR	0.23	0.06	0.12	0.35	0.20	3.88	<0.001
		RES ⇒ ENG	0.36	0.11	0.15	0.56	0.24	3.36	<0.001
	Direct	EI ⇒ AR	0.18	0.11	−0.04	0.40	0.13	1.64	0.102
		RES ⇒ AR	−0.26	0.13	−0.52	0.00	−0.15	−1.95	0.051
	Total	EI ⇒ AR	0.25	0.11	0.03	0.46	0.17	2.20	0.028
		RES ⇒ AR	−0.18	0.13	−0.44	0.09	−0.10	−1.32	0.187

Note. EI = Emotional intelligent; RES = Resilience; ENG = Engagement; SWL = Satisfaction with Life; AR = Academic Record.

## Data Availability

The data are available for anyone who wants to see them with justified reasons. Please contact the correspondence author.
